# Supplementary material for: The genome sequence of the Triangle-marked Roller, Ancylis achatana (Denis & Schiffermüller), 1775
Source: Wellcome Open Res. 2025 Mar 14;10:128. [Version 1] doi: 10.12688/wellcomeopenres.23897.1 (PMC12485347; doi:10.12688/wellcomeopenres.23897.1)
Supplement: Peer review history

# Reviewer report: Reviewer response for version 1

**DOI:** 10.21956/wellcomeopenres.26364.r121273

**Published:** 2025-05-13

**Authors:** Ricardo Rodriguez-de-la-vega

**Affiliations:** 1 Université Paris-Saclay, Gif-sur-Yvette, France

In this genome note, the authors report the reference-quality, cobiont-free genome assembly of a male specimen of Ancylis achatana, a small moth distributed across Europe and the islands of Britain and Ireland. This genome represents the first assembly from the genus Ancylis, the 35th Tortricidae, and the 487th Lepidoptera genome released as part of the Darwin Tree of Life (DToL) project as of April 12th, 2025. The report follows the standard DToL genome note format, providing core metadata and quality metrics relevant for future research and comparative genomics.

 The genome was assembled using high-coverage PacBio HiFi reads scaffolded with Hi-C data, resulting in a highly contiguous assembly, a chromosome-sized scaffold N50 and assembly pseudomolecules almost entirely assigned to chromosomal-level scaffolds. The assembly shows excellent completeness with a BUSCO score of 98.5% (based on the Lepidoptera reference set) and no detectable contamination, which places it among the highest-quality assemblies produced by DToL to date. The Hi-C contact map also indicates a successful chromosomal reconstruction.

 One suggestion for improvement would be to include links to the Tree of Life quality control (ToLQC) portal and to the European Nucleotide Archive (ENA) entries associated with this assembly, which are standard and useful resources for researchers seeking to access the raw data and quality assessments. I am including them below so readers can access them directly from the report. 

https://www.ebi.ac.uk/ena/browser/view/GCA_964214005.1

https://tolqc.cog.sanger.ac.uk/darwin/insects/Ancylis_achatana/

 As of now, the genome does not appear to be included in the Genomes on a Tree (GoaT) portal, which would facilitate phylogenomic context and visualization once integrated.

 In summary, this genome note provides a high-quality reference for A. achatana, an little studied moth lineage, and is a valuable addition to the growing Lepidoptera genomic resources. With the addition of direct data repository links, the note would better support downstream reuse and transparency.

Are sufficient details of methods and materials provided to allow replication by others?

Yes

Is the rationale for creating the dataset(s) clearly described?

Yes

Are the datasets clearly presented in a useable and accessible format?

Yes

Are the protocols appropriate and is the work technically sound?

Yes

Reviewer Expertise:

Comparative genomics,  bioinformatics,  evolution

I confirm that I have read this submission and believe that I have an appropriate level of expertise to confirm that it is of an acceptable scientific standard.

# Reviewer report: Reviewer response for version 1

**DOI:** 10.21956/wellcomeopenres.26364.r121274

**Published:** 2025-05-07

**Authors:** Arun Arumugaperumal

**Affiliations:** 1 Department of Biotechnology, Rajalakshmi Engineering College, Thandalam, Chennai, 602105, India

The data note describes the whole genome sequencing of *Ancylis achatana*, known as Triangle-marked Roller. This is the first report of the high quality genome sequence of this species. The authors have followed high-end methodology to sequence the DNA. The assembly was of size 547.27 Mb. The 16 kb mitogenome was also presented. 

 The photograph used for figure 1 is not clear. Kindly replace with a more-clear photograph, if available. All the NCBI accessions provided are working fine.

Are sufficient details of methods and materials provided to allow replication by others?

Yes

Is the rationale for creating the dataset(s) clearly described?

Yes

Are the datasets clearly presented in a useable and accessible format?

Yes

Are the protocols appropriate and is the work technically sound?

Yes

Reviewer Expertise:

Bioinformatics, Genomics

I confirm that I have read this submission and believe that I have an appropriate level of expertise to confirm that it is of an acceptable scientific standard.

# Reviewer report: Reviewer response for version 1

**DOI:** 10.21956/wellcomeopenres.26364.r121276

**Published:** 2025-04-29

**Authors:** Bryan Brunet

**Affiliations:** 1 Ottawa Research and Development Centre, Ontario, Canada

The manuscript entitled “The genome sequence of the Triangle-marked Roller, *Ancylis achatana *(Denis & Schiffermüller), 1775” by authors Boyes et al presents a chromosome length genome assembly for the tortricid leaf roller moth, *Ancylis achatana*. A combination of PacBio HiFi long reads and Arima v2 HiC chromosome conformation capture technology was used, resulting in 28 chromosome length pseudomolecules and an additional 83 unplaced scaffolds, in addition to a complete mitochondrial genome. The number of chromosome scaffolds and the size of the genome approximates the expected number of chromosomes from related tortricids with genome assemblies that are publicly available. Assembly metrics are good and the approaches used are standard approaches employed by and publicly available by the Wellcome Sanger Institute’s Tree of Life projects. The authors are encourage to consider including an annotated mitogenome assembly as a separate figure, since annotations for the mitogenome are already known from the MitoHIFI output.

 Other suggested edits to improve clarity around the methods are requested below:

 -The relevance/importance of a genome assembly for this species should be elaborated.

 -Reporting the size of the mitogenome as 0.02 Mb in Table 3 is uninformative

 -Under the PacBio Hifi section, “samples” and “libraries” are referred to, but the sample collection information indicates that a single specimen was used.

 -Under the Assembly section, the authors map Arima Hi-C reads to the assembly but do not mention the use of the recommended Arima mapping pipeline; this pipeline is meant to remove chimerism, low quality reads, and merge technical replicates before removing pcr duplicates – please explain why it wasn’t used and if/how these steps were performed.

 -Provide a reference for FastK

 -How was the synteny analysis conducted?

 -The two paragraphs on the blobtoolkit pipeline seem disconnected from the rest of the section (ie. there’s no introduction to what the toolkit is being used for - even if most of the readership probably already knows, this should be stated). Also, the second paragraph gives the impression that the blobtoolkit pipeline (as used via nextflow: sanger-tol/blobtoolkit v0.7.1) was newly developed, but that’s not the case. I believe the appropriate citation is Zaynab et al. 2025

 -Which versions of uniprot and nt dbs?

 -ASCC pipeline reference under Assembly curation, needs a reference.

 -Table 4 is missing genomescope, purge_dups, mitofinder, ASCC pipeline, JBrowse2, singularity

Are sufficient details of methods and materials provided to allow replication by others?

Partly

Is the rationale for creating the dataset(s) clearly described?

Partly

Are the datasets clearly presented in a useable and accessible format?

Yes

Are the protocols appropriate and is the work technically sound?

Yes

Reviewer Expertise:

hemiptera systematics and taxonomy, genomics

I confirm that I have read this submission and believe that I have an appropriate level of expertise to confirm that it is of an acceptable scientific standard, however I have significant reservations, as outlined above.

# Reviewer report: Reviewer response for version 1

**DOI:** 10.21956/wellcomeopenres.26364.r121278

**Published:** 2025-03-28

**Authors:** Kay Lucek

**Affiliations:** 1 University of Neuchâtel, Neuchâtel, Switzerland

The authors present the chromosome level genome assembly of a male specimen of the Triangle-marked Roller moth ( *Ancylis achatana*). The assembly consists of 28 chromosomes including the Z chromosome. The assembly is highly complete as revealed by the high BUSCO score but not fully phased. Sequencing and genome assembly follow the current state of the art and use established methods.

 Overall, the presented assembly will be of great value to study the evolution of Lepidoptera through comparative studies and shed light on the diversity of this less well studied branch of the Tree of Life. Interestingly the Z chromosome was identified through synteny analyses with the distantly related *Archips crataeganus*. It would have been nice to give more details why this species was used in particular and how the synteny analysis was performed.

Are sufficient details of methods and materials provided to allow replication by others?

Yes

Is the rationale for creating the dataset(s) clearly described?

Yes

Are the datasets clearly presented in a useable and accessible format?

Yes

Are the protocols appropriate and is the work technically sound?

Yes

Reviewer Expertise:

Speciation genomics

I confirm that I have read this submission and believe that I have an appropriate level of expertise to confirm that it is of an acceptable scientific standard.
